# Target Serum Urate Achievement and Chronic Kidney Disease Progression in Patients With Gout and Kidney Disease

**DOI:** 10.1001/jamainternmed.2024.6212

**Published:** 2024-11-25

**Authors:** Yilun Wang, Nicola Dalbeth, Robert Terkeltaub, Yuqing Zhang, Xiaoxiao Li, Chao Zeng, Guanghua Lei, Jie Wei

**Affiliations:** 1 Department of Orthopaedics, Xiangya Hospital, Central South University, Changsha, China; 2 Department of Medicine, University of Auckland, Auckland, New Zealand; 3 University of California at San Diego, La Jolla, San Diego; 4 Division of Rheumatology, Allergy, and Immunology, Department of Medicine, Massachusetts General Hospital, Harvard Medical School, Boston; 5 The Mongan Institute, Massachusetts General Hospital, Harvard Medical School, Boston; 6 Hunan Key Laboratory of Joint Degeneration and Injury, Xiangya Hospital, Central South University, Changsha, China; 7 Key Laboratory of Aging-Related Bone and Joint Diseases Prevention and Treatment, Ministry of Education, Xiangya Hospital, Central South University, Changsha, China; 8 National Clinical Research Center for Geriatric Disorders, Xiangya Hospital, Central South University, Changsha, China; 9 Department of Epidemiology and Health Statistics, Xiangya School of Public Health, Central South University, Changsha, China

## Abstract

**Importance:**

Clinicians often approach urate-lowering therapy (ULT) cautiously in patients with gout and impaired kidney function because they are concerned about the risk of progression to severe or end-stage kidney disease. However, evidence from randomized clinical trials of this association remains inconclusive.

**Objective:**

To evaluate the association between achieving target serum urate level with ULT and progression of chronic kidney disease (CKD) to severe or end-stage in patients with gout and impaired kidney function.

**Design, Setting, and Participants:**

This was a cohort study using the target trial emulation approach using data from a general practice database (IQVIA Medical Research Database) for 2000 to 2023. Eligible patients were 40 to 89 years old and had gout and CKD stage 3. Data analyses were performed from November 2023 to September 2024.

**Exposures:**

Lowering serum urate level to target level (<6 mg/dL) using ULT.

**Main Outcomes and Measures:**

Severe or end-stage kidney disease, determined by an estimated glomerular filtration rate of less than 30 mL/min/1.73 m^2^ on at least 2 occasions more than 90 days apart within 1 year, or at least 1 Read code (per the Refined Etiology, Anatomical Site, and Diagnosis classification) for CKD stages 4 or 5, hemodialysis, peritoneal dialysis, or kidney transplant. The prespecified noninferiority margin of the hazard ratio (HR) was set at 1.2, comparing those who achieved the target serum urate level with those who did not.

**Results:**

Among the 14 792 participants (mean [SD] age, 73.1 [9.5] years; 9215 men [62.3%] and 5577 women [37.7%]) with gout and with CKD stage 3, the 5-year risk of severe or end-stage kidney disease was 10.32% for those who achieved the target serum urate level and 12.73% for those who did not. Compared with those not achieving the target level, the adjusted 5-year risk difference and HR of severe or end-stage kidney disease for patients achieving the target serum urate level was −2.41% (95% CI, −4.61% to −0.21%) and 0.89 (95% CI, 0.80 to 0.98), respectively.

**Conclusions and Relevance:**

The findings of this cohort study indicate that in patients with gout and CKD stage 3, lowering serum urate level to less than 6 mg/dL vs 6 mg/dL or greater using ULT was not associated with an increased risk of severe or end-stage kidney disease. These findings support optimizing ULT to achieve target serum urate levels when treating patients with gout and impaired kidney function.

## Key Points

**Question:**

How does urate-lowering therapy (ULT) affect the progression of chronic kidney disease (CKD) in patients with gout and impaired kidney function?

**Findings:**

This cohort study including 14 792 patients with gout and CKD stage 3 found that lowering serum urate level to less than 6 mg/dL using ULT was not associated with a higher risk of severe or end-stage kidney disease progression compared with those who did not achieve this level with ULT.

**Meaning:**

This finding suggests that achieving serum urate levels less than 6 mg/dL with ULT is well tolerated in patients with gout and CKD stage 3; therefore, optimizing ULT use could benefit both clinicians and patients.

## Teaser

This cohort study evaluates the use of urate-lowering therapy to achieve target serum urate levels in patients with gout and stage 3 chronic kidney disease.

## Introduction

Gout is a common chronic inflammatory arthritis that affects 1% to 5% of adults worldwide,^1,2^ and projections indicate a continued increase in the coming years.^3^ Gout flares are associated with severe pain, poor quality of life, and a transient increase in major cardiovascular and venous thrombotic events.^4,5,6^ Given that hyperuricemia is a known causal risk factor for gout,^7^ urate-lowering therapy (ULT) is the cornerstone of long-term management. Experts propose optimal ULT administration as a remission-inducing measure for gout,^8^ and treating to a serum urate target of less than 6 mg/dL (to convert to mmol/L, multiply by 0.0595) has successfully reduced gout flares over the long-term and improved overall outcomes in recent trials.^9,10,11^ Rheumatology societies, such as the American College of Rheumatology and the European Alliance of Associations for Rheumatology, endorse this treat-to-target approach as best practice.^12,13^ Despite these recommendations, approximately half of the patients with gout receive ULT and achieve the target serum urate level (TSUL) in clinical general practice, where most patients with gout are treated, remains below 30%.^11,14,15,16^ Several factors, including lack of monitoring and treatment interruptions, may contribute to this discrepancy.^15,17^

The discrepancy is further compounded by the frequent comorbidity of chronic kidney disease (CKD), which is present in 20% to 30% or more of patients with gout.^18^ Hyperuricemia is associated with a higher risk of kidney disease^19,20,21^; however, there is a lack of conclusive evidence on whether ULT may worsen kidney function among patients with gout and CKD. Observational studies^22,23,24^ and randomized clinical trials (RCTs)^25,26,27^ conducted in individuals with hyperuricemia and impaired kidney function have shown protective effects against CKD progression. Three RCTs reported no apparent difference in kidney function decline between ULT and placebo among patients with impaired kidney function.^28,29,30^ However, these trials included participants with CKD but without gout,^28^ type 1 diabetes and diabetic kidney disease regardless of gout status,^29^ and CKD with hyperuricemia^30^; none specifically focused on patients with gout. Only 1 RCT assessed ULT among patients with gout with moderate to severe kidney function impairment, and the results did not show a significant difference in kidney function between febuxostat and placebo groups.^31^ However, it did not assess whether lowering serum urate to a target level (ie, <6 mg/dL), as recommended by professional rheumatology societies, was associated with the risk of CKD progression.

Thus, there is no clear evidence that ULT worsens kidney function in patients with gout with CKD. Despite this, some clinicians opt to withhold, reduce, or even discontinue ULT when a patient with gout experiences a decline in kidney function, complicating gout management.^32^ This practice, often reported anecdotally by rheumatologists who note that “nephrologists keep lowering my patient’s allopurinol for no reason,”^33^ contributes to public health concerns regarding the potential risks of ULT, including the rare but serious risk of allopurinol hypersensitivity syndrome.^34^ Addressing this issue is crucial for the timely and effective management of CKD progression and gout.

We conducted a cohort study emulating RCTs to evaluate the association between achieving TSUL with ULT and the risk of severe or end-stage kidney disease progression in patients with impaired kidney function who developed gout.

## Methods

This study was reviewed and approved by the The Health Improvement Network (THIN) Scientific Review Committee (21SRC003-A2) and the medical ethical committee at Xiangya Hospital (2018091077). The IQVIA Medical Research Database (IMRD) research use is approved by the UK National Health Service Health Research Authority (NHS Research Ethics Committee EM/0151). Informed consent was waived because the data provided to the researchers were deidentified. The study followed the strengthening the Reporting of Observational Studies in Epidemiology (STROBE) reporting guideline.

### Data Source

We used data from the IMRD incorporating data from THIN, a Cegedim database. IMRD contains longitudinal, nonidentified patient electronic health care records collected from general practitioner (GP) clinical systems in the United Kingdom. Specific diagnoses are coded using the Read classification system,^35^ whereas drugs are coded using a dictionary based on the Multilex classification system. The validity of IMRD for clinical and epidemiologic research has been well established.^36,37^

### Study Participants 

**Figure 1.**
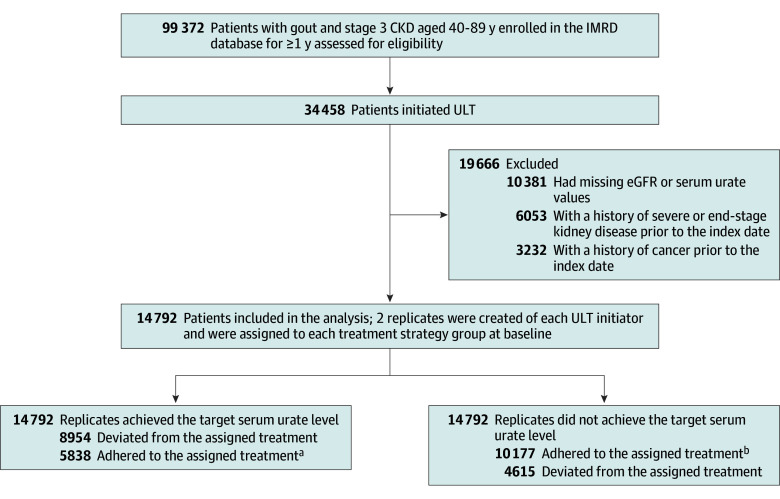
Flow Diagram of the Included Participants CKD indicates chronic kidney disease; eGFR, estimated glomerular filtration rate; IMRD, the IQVIA Medical Research Database; TSUL, target serum urate level; and ULT, urate-lowering therapy. ^a^The 5838 replicates adhered to the assigned treatment included those who achieved TSUL and encountered an incident of severe or end-stage kidney disease, loss to follow-up, or death during the first year of follow-up. ^b^The 10 177 replicates adhered to the assigned treatment included those who did not achieve TSUL and encountered an incident of severe or end-stage kidney disease, loss to follow-up, or death before achieving TSUL during the first year of follow-up.

We included participants aged 40 to 89 years with gout and CKD stage 3 between January 1, 2000, and June 30, 2023, who had at least 1 year of continuous GP enrollment before entering the study. Gout was defined using Read codes based on previous studies using IMRD.^38,39,40^ CKD stage 3 was defined as either an estimated glomerular filtration rate (eGFR) from 30 to 60 mL/min/1.73 m^2^ on at least 2 occasions more than 90 days apart within 1 year or at least 1 Read code for CKD stage 3.^40^ ULT initiators were identified by their first ULT prescription after gout and CKD stage 3 diagnosis, with the prescription date set as the index date. Individuals were excluded if they had cancer, severe or end-stage kidney disease, or lacked serum urate and eGFR measurement data before the index date. There were 34 458 eligible patients who met the inclusion criteria and initiated ULT during the study period (Figure 1).

### Study Design

**Figure 2.**
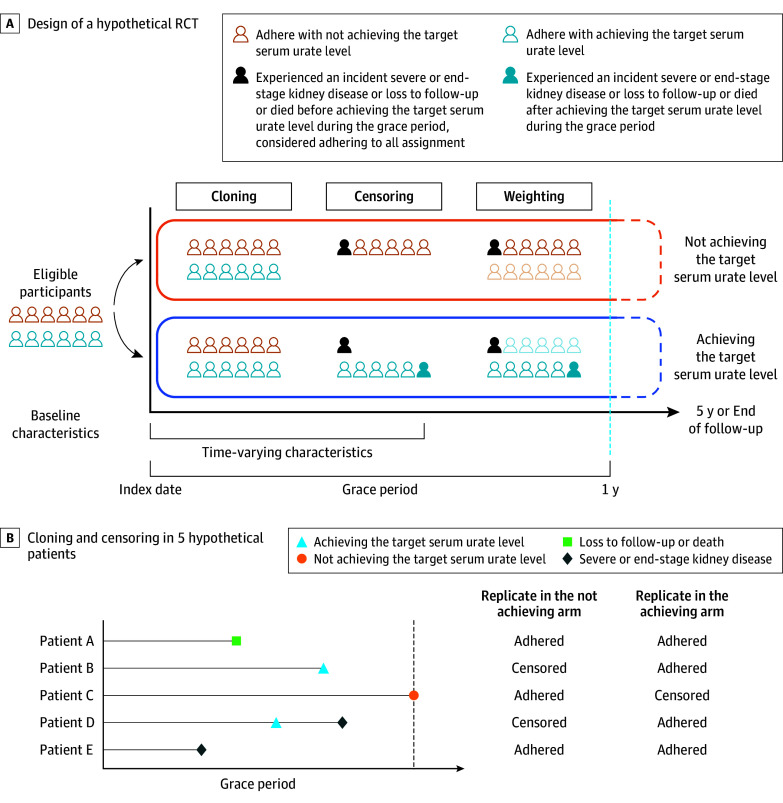
Study Design RCT indicates randomized clinical trial.

We emulated analyses of a hypothetical target trial using a cloning, censoring, and weighting approach to assess the association between achieving the target level of less than 6 mg/dL within 1 year after initiation of ULT and the risk of CKD progression (Figure 2A).^40,41,42,43^

#### Cloning

Cloning refers to assigning patients to a treatment strategy at time zero. We created 2 replicates for each participant at baseline and assigned these replicates to 1 of the 2 intervention arms: achieving TSUL or not achieving TSUL. This approach closely mirrors the randomization process in a traditional RCT.

#### Censoring

Censoring refers to ensuring that replicates followed their assigned strategy during the first year follow-up. We allowed a 1-year grace period after initiation of ULT for individuals to achieve TSUL. Replicates assigned to the arm achieving TSUL were censored if they did not achieve TSUL by the end of the grace period. Conversely, replicates assigned to the not achieving TSUL arm were censored if they achieved TSUL during the grace period. During the grace period, if an individual experienced an incident of severe or end-stage kidney disease, loss to follow-up, or death before achieving TSUL, they were handled differently depending on whether they reached TSUL (Figure 2B).

#### Selection Bias−Adjusted Weighting

Because censoring may lead to potential selection bias, we used inverse probability weights (IPW) to account for censoring.^43^ The denominator of the IPW was the probability that a replicate adhered to their assigned treatment, estimated using the logistic regression with baseline covariates and the time-varying covariates. The weight assignment for each replicate is shown in eTable 1 in Supplement 1. A detailed description of the method is provided in the eMethods in Supplement 1.

### Assessment of Outcomes

The outcome was a combination of severe or end-stage kidney disease over 5 years after the index date. Severe or end-stage kidney disease was defined as an eGFR less than 30 mL/min/1.73 m^2^ on at least 2 occasions more than 90 days apart within 1 year or at least 1 Read code for CKD stages 4 or 5, hemodialysis, peritoneal dialysis, or kidney transplant.^40^

### Assessment of Covariates

Baseline covariates included sociodemographic factors (age, sex, and socioeconomic deprivation index score), body mass index (BMI), lifestyle factors (smoking status and drinking status), serum urate level, comorbidities (atrial fibrillation, chronic obstructive pulmonary disease, dementia, depression, diabetes, fall, fracture, hypertension, myocardial infarction, osteoporosis, pneumonia or infection, stroke, varicose veins, and venous thromboembolism) before the index date, medication use (nonsteroidal anti-inflammatory drugs [NSAIDs], opioids, antihypertensive, antidiabetic, nitrates, aspirin, anticoagulants, and colchicine), and health care utilization (number of hospitalizations, GP visits, and referrals from specialists) during the year before the index date. Serum creatinine levels were obtained from the database before the index date. The eGFR was calculated using serum creatinine levels via the Modification of Diet in Renal Disease formula.^44^ Time-varying covariates included BMI, lifestyle factors, comorbidities, and medication use between the index date and the censoring date.

### Statistical Analysis

Participants were followed up until the first occurrence of severe or end-stage kidney disease, death, disenrollment from a GP practice participating in IMRD, 5 years of follow-up, or at the end of the study (June 30, 2023). The follow-up time was divided into 5 single year time blocks starting from ULT initiation. To control for the competing risk of death, we performed a cross-sectional pooling analysis to estimate the odds ratio and its corresponding 95% CIs,^45^ including an indicator for achieving TSUL and adjusting for the year of follow-up (linear and quadratic term) and baseline covariates in the weighted population. The odds ratio estimated from this model approximated the hazard ratio (HR) because the outcome is rare. We used robust standard errors to calculate a 95% CI for HR. We estimated the 5-year absolute risk difference of severe or end-stage kidney disease by fitting the pooled logistic models with product terms between the indicator for achieving TSUL and the year of follow-up variables. A nonparametric bootstrap analysis with 100 samples was used to compute the 95% CIs for absolute estimates. We also assessed the association of achieving TSUL with the risk of end-stage kidney disease alone, defined as an eGFR less than 15 mL/min/1.73 m^2^ on at least 2 occasions more than 90 days apart within 1 year, or at least 1 Read code for CKD stage 5, hemodialysis, peritoneal dialysis, or kidney transplant.^40^ To handle missing covariate data, we used multiple imputations by chained equations and applied Rubin rules to combine results across the 5 imputed datasets in sensitivity analysis.^46^

To further assess the robustness of our findings, we took the same approach to evaluate the association between achieving TSUL and the risk of severe or end-stage kidney disease, as well as end-stage kidney disease alone, among patients with gout and CKD stages 2 to 3. Patients with CKD stage 2 were defined by an eGFR between 60 and 90 mL/min/1.73 m^2^ on at least 2 occasions more than 90 days apart within 1 year or at least 1 Read code for CKD stage 2.

Based on evidence from previous RCTs,^28,29^ we set the noninferiority margin at 1.2 for the HR when comparing individuals who lowered their serum urate levels to 6 mg/dL with those whose serum urate levels remained higher than 6 mg/dL. If the upper boundary of the 95% CI for the HR was less than the noninferiority margin, then noninferiority was established, and a 1-sided α of .025 was considered as the threshold for statistical significance for the tests. All analyses were conducted using SAS software, version 9.4 (SAS Institute), from November 2023 to September 2024.

## Results

The primary analysis included 14 792 participants (mean [SD] age, 73.1 [9.5] years; 9215 men [62.3%] and 5577 women [37.7%]). The mean (SD) serum urate and eGFR values were 8.9 (1.6) mg/dL and 49.9 (12.3) mL/min/1.73 m^2^, respectively (Table 1). Baseline characteristics and time-varying covariates were well balanced between the 2 comparison groups after IPW (eTables 2 and 3 in Supplement 1). Additionally, among these ULT initiators, 14 615 (98.8%) were prescribed allopurinol and 177 (1.2%) received febuxostat.

**Table 1.** Baseline Characteristics of Included Participants

Characteristic	Study population (N = 14 792), %
Demographic information	
Age, mean (SD), y	73.1 (9.5)
SDI, mean (SD)^a^	2.5 (1.5)
Female, No. (%)	5577 (37.7)
Male, No. (%)	9215 (62.3)
BMI category	
Normal	14.2
Obese	37.6
Overweight	44.2
Underweight	0.3
Missing data	3.7
Serum urate, mean (SD), mg/dL	8.9 (1.6)
eGFR, mean (SD), mL/min/1.73 m^2^	49.9 (12.3)
Lifestyle factors	
Drinking alcohol	
None	18.5
Past	3.4
Current	73.2
Missing	4.9
Smoking tobacco	
None	48.6
Past	43.9
Current	7.0
Missing	0.5
Comorbidity	
Atrial fibrillation	22.0
Chronic obstructive pulmonary disease	10.0
Dementia	1.3
Depression	10.5
Diabetes	26.5
Fall	14.0
Fracture	1.1
Hypertension	79.7
Myocardial infarction	14.6
Osteoporosis	5.3
Pneumonia or infection	9.2
Stroke	7.3
Varicose veins	9.6
Venous thromboembolism	6.0
Medication^b^	
Anticoagulants	18.0
Antidiabetic medicine	17.1
Antihypertensive medicine	92.5
Aspirin	38.6
Colchicine	49.7
Loop diuretics	40.1
Nitrates	13.2
NSAIDs	69.9
Opioids	18.1
Potassium-sparing diuretics	13.9
Thiazide diuretics	31.9
Health care utilization, mean (SD)^b^	
Hospitalizations	0.6 (1.3)
General practice visits	9.0 (7.7)
Specialist referrals	0.7 (1.2)

Abbreviations: BMI, body mass index (calculated as weight in kilograms divided by height in meters squared); eGFR, estimated glomerular filtration rate; NSAID, nonsteroidal anti-inflammatory drug; SDI, Socioeconomic Deprivation Index.

^a^ Measured by the Townsend Deprivation Index, which was grouped into quintiles from 1 (least deprived) to 5 (most deprived).

^b^ Frequency during the past year.

**Figure 3.**
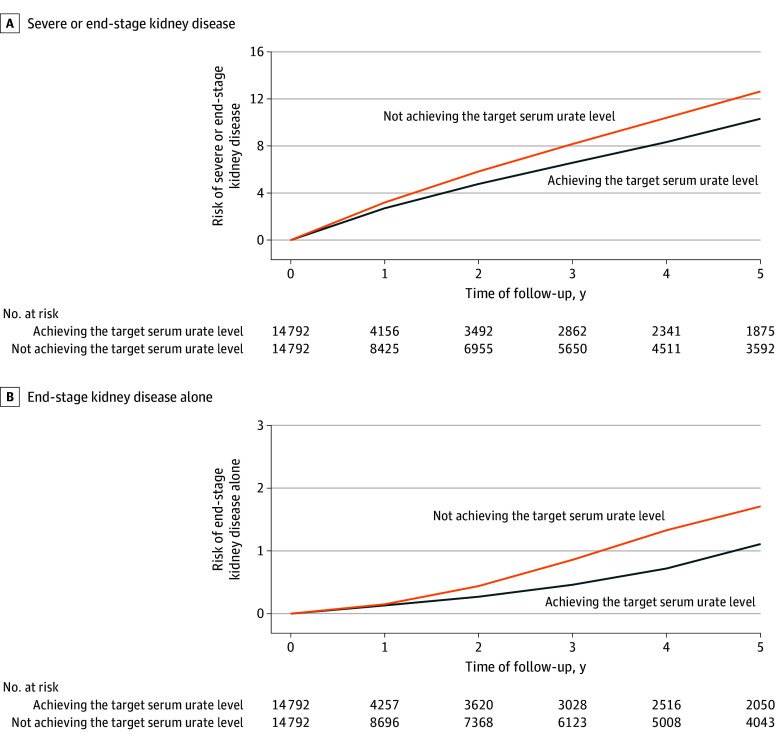
Association of Achieving Target Serum Urate Level (TSUL) and Chronic Kidney Disease (CKD) Progression in Participants With Gout and CKD Stage 3 Five-year risk of severe or end-stage kidney disease and end-stage kidney disease alone among participants with gout and CKD stage 3 achieving TSUL and not achieving TSUL with urate-lowering therapy.

Among the 14 792 ULT initiators, 4706 (31.8%) achieved TSUL within 1 year of the index date. The mean (SD) treatment duration of ULT was 243.5 (135.7) days in the achieving TSUL arm and 206.4 (151.9) days in the not achieving TSUL arm. As shown in Figure 3A, the 5-year risk of severe or end-stage kidney disease was not inferior in the achieving TSUL arm (10.32%) than in the not achieving TSUL arm (12.73%). The adjusted risk difference was −2.41% (95% CI, −4.61% to −0.21%), and the adjusted HR was 0.89 (95% CI, 0.80 to 0.98) (Table 2). The upper boundary of the 95% CI for the HR is below the prespecified noninferiority margin. The imputation of missing data resulted in similar findings (adjusted HR, 0.91; 95% CI, 0.81 to 0.99; *P* <.001 for noninferiority). Comparable results were also observed when the association of lowering serum urate with ULT to the target level with the risk of end-stage kidney disease alone was assessed (Figure 3B and Table 2).

**Table 2.** Association of Achieving Target Serum Urate Level (TSUL) Induced by Urate-Lowering Therapy to Chronic Kidney Disease (CKD) Progression in Patients With Gout and CKD Stage 3

CKD progression	Not achieving TSUL	Achieving TSUL
Total participants, No.	14 792	14 792
**Severe to end-stage kidney disease, weighted**		
Cases, No. (%)	1542 (10.4)	1030 (7.0)
Follow-up, mean (SD) y	3.2 (2.1)	3.4 (2.5)
Risk over 5 years, %	12.73	10.32
Risk difference, % (95% CI)	0 [Reference]	−2.41 (−4.61 to −0.21)
HR (95% CI)	1.00 [Reference]	0.89 (0.80 to 0.98)^a^
HR from imputation (95% CI)	1.00 [Reference]	0.91 (0.81 to 0.99)^a^
**End-stage kidney disease alone, weighted**		
Cases, No. (%)	184 (1.2)	88 (0.6)
Follow-up, mean (SD) y	3.3 (2.1)	3.5 (2.5)
Risk over 5 years, %	1.73	1.10
Risk difference, % (95% CI)	0 [Reference]	−0.63 (−0.94 to −0.32)
HR (95% CI)	1.00 [Reference]	0.67 (0.46 to 0.97)^b^
HR from imputation (95% CI)	1.00 [Reference]	0.76 (0.52 to 1.00)^a^

Abbreviation: HR, hazard ratio.

^a^ *P* for noninferiority <.001.

^b^ *P* for noninferiority = .001.

Consistent findings were observed when analyses were performed on participants with gout and CKD stages 2 to 3. Of the 31 066 ULT initiators with gout and CKD stages 2 to 3, 10 514 (33.8%) achieved TSUL within 1 year after the index date. As shown in eTable 4 and the eFigure in Supplement 1, the 5-year risks of severe or end-stage kidney disease (adjusted HR, 0.91; 95% CI, 0.82 to 1.00) and end-stage kidney disease alone (adjusted HR, 0.73; 95% CI, 0.52 to 1.04) in the achieving TSUL arm were not inferior compared with the not achieving TSUL arm, with the *P* for noninferiority <.001 and .003, respectively. Similar trends were observed when missing data were imputed.

## Discussion

In this large population-based GP database, lowering serum urate level to less than 6 mg/dL among patients with gout and CKD stage 3 was not associated with a higher risk of severe or end-stage kidney disease progression than not achieving this level with ULT. Similar results were observed among participants with gout and CKD stages 2 to 3. These findings suggest that achieving TSUL with ULT does not worsen kidney function in patients with gout and moderate CKD.

### Comparison With Previous Studies

Previous observational studies^22,23,24^ and RCTs^25,26,27^ have reported that ULT reduced CKD progression risk, while other RCTs have found no such effect, particularly in patients without gout.^28,29,30,47^ Given the inherent differences between individuals with gout and those with asymptomatic hyperuricemia, the findings from these studies may not be directly applicable to patients with gout.^34^ Only 1 RCT involving patients with gout and moderate to severe kidney impairment found no significant difference in kidney function between febuxostat and placebo.^31^ However, this study did not assess the effect of lowering serum urate to a target level, an indicator of successful gout management, on the risk of CKD progression. Our study observed that lowering serum urate to less than 6 mg/dL within 1 year after initiating ULT among patients with gout and CKD stage 3 was not associated with an increased risk of severe or end-stage kidney disease. These results were consistent with the findings from several large RCTs, suggesting ULT does not have any significant harmful effect on kidney function among patients with CKD, regardless of gout.

### Possible Explanations

Proposed biological mechanisms suggest a potential association between ULT and kidney function. First, elevated urate may increase glomerular hydrostatic pressure by stimulating vascular smooth muscle cell proliferation in afferent arterioles, resulting in associated vessel stiffening, loss of autoregulation, and glomerular hypertension.^48^ Consequently, by reducing serum urate levels, ULT may diminish glomerular hydrostatic pressure, thereby mitigating kidney damage. Second, crystal deposition has proinflammatory properties and can induce the synthesis of inflammatory cytokines, contributing to the development of vascular diseases and atherosclerosis.^49,50,51^ Lowering serum urate to target levels could exert anti-inflammatory effects, serving as an alternative therapeutic strategy to address endothelial dysfunction, a modifiable factor in the CKD progression.^52^ This hypothesis may explain why some RCTs of patient without gout, who may lack kidney tissue uric acid crystals, have not observed the expected effects of ULT on kidney function.^28,29^ Third, during the catalysis of urate production, xanthine oxidase generates excessive reactive oxygen species, which can damage glomerular endothelial cells and impair endothelial function.^53^ Therefore, xanthine oxidase inhibitors can help preserve glomerular endothelial function by reducing reactive oxygen species production and restoring compromised glomerular permeability, thereby mitigating the deterioration of kidney function.^54,55^

### Clinical and Research Implications

Kidney impairment is common among patients with gout and has historically presented substantial challenges to effective gout management.^56^ Our study contributes to the limited literature on therapeutic strategies for patients with gout and impaired kidney function. Our findings suggest that lowering serum urate levels to less than 6 mg/dL is generally well tolerated and may even slow CKD progression in these individuals. Initiatives to optimize the use and adherence to ULT could benefit clinicians and patients. Furthermore, our study raises an important question about whether the risk-benefit assessment of a treat-to-target approach with ULT should be reevaluated for patients with gout and CKD in light of this evidence. Although ULT is generally well tolerated, it has been associated with rare but severe hypersensitivity reactions, such as allopurinol hypersensitivity syndrome.^57,58^ If future studies corroborate our findings, reassessing the risk-benefit ratio for using ULT in patients with gout and CKD may be warranted.

### Strengths and Limitations

Using a clinical population-based database, we emulated an RCT to compare the risk of CKD progression in patients with gout and impaired kidney function who achieved TSUL through ULT compared with those who did not. This approach enabled us to evaluate the impact of hyperuricemia on the increased risk of CKD progression in patients with gout and impaired kidney function while mitigating potential biases such as selection bias (initiators of ULT) and confounding by indication (all participants received ULT).

This study had some limitations of note. Although we used rigorous methods to control confounders by emulating an analysis of RCT, residual confounding cannot be ruled out given that it is common in observational studies. Second, ULT initiators who achieved TSUL may have received better health care, adhered more consistently to other treatments, and used ULT longer than their comparators. This made it challenging to separate the effects of health care quality and adherence to other treatment recommendations from the impact of lowering urate levels. In our analysis, we adjusted for both baseline covariates and time-varying covariates from the index date to the censoring date. Importantly, no differences were observed between the 2 groups regarding health care utilization before ULT initiation or in the time-varying covariates between initiation and censoring among individuals who achieved the TSUL and those who did not. This suggests that the effect of health care utilization and adherence to other treatment recommendations on the risk of CKD progression, if it exists, may not completely explain the results found in our study. In addition, excluding participants who did not have the serum urate level before the index date may limit the generalizability of the current findings to a population that may receive less health care or experience less severe disease.

## Conclusions

This cohort study found that among patients with gout and CKD stage 3, lowering serum urate level with ULT to less than 6 mg/dL vs 6 mg/dL or greater was not associated with an increased risk of severe or end-stage kidney disease. These findings support optimizing ULT to achieve TSUL when treating patients with gout and impaired kidney function.

## Notes

Supplement 1. eMethods eTable 1. Censoring weights eTable 2. Baseline characteristics between those who achieved the TSUL through ULT in individuals with gout and CKD stage 3 and those who did not eTable 3. Time-varying covariates between those who achieved the TSUL through ULT in individuals with gout and CKD stage 3 and those who did not eTable 4. Relationship of achieving TSUL induced by ULT to CKD progression in patients with gout and CKD stages 2-3 eFigure. Association of achieving TSUL achievement and CKD progression in gout patients with CKD stage 2-3

Supplement 2. Data Sharing Statement
