# Supplementary material for: Target Serum Urate Achievement and Chronic Kidney Disease Progression in Patients With Gout and Kidney Disease
Source: JAMA Intern Med. 2024 Nov 25;185(1):74–82. doi: 10.1001/jamainternmed.2024.6212 (PMC11589860; doi:10.1001/jamainternmed.2024.6212)
Supplement: Supplement 1. — eMethods eTable 1. Censoring weights eTable 2. Baseline characteristics between those who achieved the TSUL through ULT in individuals with gout and CKD stage 3 and those who did not eTable 3. Time-varying covariates between those who achieved the TSUL through ULT in individuals with gout and CKD stage 3 and those who did not eTable 4. Relationship of achieving TSUL induced by ULT to CKD progression in patients with gout and CKD stages 2-3 eFigure. Association of achieving TSUL achievement and CKD progression in gout patients with CKD stage 2-3 [file jamainternmed-e246212-s001.pdf]

Supplemental Online Content

Wang Y, Dalbeth N, Terkeltaub R, et al. Target Serum Urate Achievement and Chronic Kidney Disease Progression in Patients With Gout and Kidney Disease. *JAMA Intern Med.* Published online November 25, 2024. doi:10.1001/jamainternmed.2024.6212

eMethods

eTable 1. Censoring weights

eTable 2. Baseline characteristics between those who achieved TSUL through ULT in individuals with gout and CKD stage 3 and those who did not

eTable 3. Time-varying covariates between those who achieved TSUL through ULT in individuals with gout and CKD stage 3 and those who did not

eTable 4. Relationship of achieving TSUL induced by ULT to CKD progression in patients with gout and CKD stages 2-3

eFigure. Association of achieving TSUL achievement and CKD progression in gout patients with CKD stage 2-3

This supplemental material has been provided by the authors to give readers additional information about their work.

Abbreviations: TSUL, target serum urate level; ULT, urate-lowering therapy.

© 2024 Wang Y et al. *JAMA Intern Med.*

eMethods

Detail information of the study design: “cloning, censoring, and weighting”

Cloning: assign patients to a treatment strategy at time zero
We assigned each individual to one of two treatment strategies: “achieving TSUL” or “not achieving TSUL” within one year after the initiation of ULT. This approach is conceptually equivalent to creating two replicates (or “clones”) of each individual in the dataset, with each replicate assigned to a different strategy. This “cloning” process ensures the two comparison groups are compatible at baseline, closely mimicking the randomization process in a traditional randomized controlled trial (RCT).

Censoring: ensure that replicates follow their assigned strategy during the first-year follow-up
To ensure that replicates adhered to their assigned strategy during the first year of follow-up, we allowed individuals a one-year grace period from baseline to achieve TSUL. Replicates that deviated from their assigned strategy during this grace period were censored. Specifically, replicates assigned to the “achieving TSUL” arm were censored if they did not achieve TSUL by the end of the first year of follow-up. Conversely, replicates assigned to the “not achieving TSUL” arm were censored if they achieved TSUL within the first year.

During the grace period, if an individual experienced an incident of severe or end-stage kidney disease, loss to follow-up, or death before achieving TSUL, they are handled differently depending on whether they reach TSUL. Specifically,

- If these events occur **before** achieving TSUL, the individual is considered to be adhering to both assignments, and the outcome contributes to each assigned arm.
- If these events occur **after** achieving TSUL, the replicate in the “achieving TSUL” arm is followed until the event occurs, and the outcome contributes to this arm. Meanwhile, the replicate in the “not achieving TSUL” arm is censored at the time of the updated SU level measurement, and the event does not contribute to that arm.

Weighting: adjust for selection bias
To address the potential selection bias due to censoring, we used inverse probability weighting (IPW). Specifically, we estimated the probability of being censored at the end of the first year of follow-up using baseline and time-varying covariates and constructed the IPW. The denominator of the IPW was the probability that a replicate adhered to his/her assigned intervention arm using the logistic regression model that included baseline and time-varying covariates (see Assessment of covariates). IPW accounts for potential differential censoring between the two comparison arms (i.e., informative censoring) by up-weighting replicates who remained in the study and had characteristics similar to those who were censored. The weight assignments are shown in **eTable 1**.

Abbreviations: TSUL, target serum urate level; ULT, urate-lowering therapy.

© 2024 Wang Y et al. *JAMA Intern Med.*

eTable 1. Censoring weights

Time Point	Not achieving TSUL	Achieving TSUL
Grace period, year (j) j=1	1/ P_{r0}	1
After grace period, year (j) 1<j≤5	1/ P_{r0}	1/ P_{r1}

P_{r0} represents the probability that a replicate adhered to the "not achieving TSUL" arm, estimated using logistic regression with baseline and time-varying covariates.
P_{r1} represents the probability that a replicate adhered to the "achieving TSUL" arm, also estimated using logistic regression with baseline and time-varying covariates.

Abbreviation: TSUL, target serum urate level.

© 2024 Wang Y et al. *JAMA Intern Med*.

eTable 2. Baseline characteristics between those who achieved TSUL through ULT in individuals with gout and CKD stage 3 and those who did not

Characteristics	Not achieving TSUL	Achieving TSUL	SMD before weighting	Not achieving TSUL	Achieving TSUL	SMD after weighting
**Demographics**						
Age, mean (SD), y	73.1 (9.5)	73.9 (9.0)	0.091	73.2 (9.2)	73.0 (9.0)	0.024
Socioeconomic deprivation index, mean (SD)*	2.5 (1.5)	2.4 (1.5)	0.047	2.5 (1.5)	2.5 (1.5)	0.014
Female (%)	35.3	41.8	0.135	37.6	39.0	0.027
**BMI category (%)**			0.071			0.016
Normal	13.7	15.6		14.2	14.2	
Obese	37.2	38.1		37.7	37.9	
Overweight	44.9	42.2		44.2	43.8	
Underweight	0.3	0.5		0.3	0.3	
Missing	3.8	3.6		3.6	3.8	
**Serum urate, mean (SD), mg/dL**	9.1 (1.6)	8.7 (1.7)	0.240	8.9 (1.6)	8.8 (1.6)	0.067
**eGFR, mean (SD), mL/min/1.73m^2**	49.3 (12.5)	50.2 (12.1)	0.073	50.0 (14.2)	50.7 (11.6)	0.053
**Lifestyle factors**						
Drinking (%)			0.038			0.034
None	18.4	18.8		18.5	18.3	
Past	3.5	3.3		3.4	3.2	
Current	72.9	73.4		72.9	74.0	
Missing	5.2	4.4		5.2	4.5	
Smoking (%)			0.028			0.033
None	48.1	49.0		48.6	48.6	
Past	44.4	43.6		43.8	44.0	
Current	7.1	6.8		7.1	6.8	
Missing	0.5	0.6		0.5	0.7	
**Comorbidity (%)**						
Hypertension	79.3	80.1	0.020	79.9	79.9	0.001
Diabetes	27.2	26.9	0.007	26.5	25.3	0.027
Myocardial infarction	15.6	13.9	0.047	14.6	13.6	0.028
Pneumonia or infection	9.6	8.4	0.040	9.2	8.8	0.013
Dementia	1.3	1.6	0.025	1.2	1.2	0.003
Depression	10.4	11.0	0.021	10.5	10.6	0.004
Chronic obstructive pulmonary disease	10.3	9.9	0.016	10.1	9.4	0.021
Fall	14.0	15.1	0.031	14.2	13.9	0.010

© 2024 Wang Y et al. *JAMA Intern Med.*

Stroke	7.3	7.6	0.013	7.3	7.1	0.008
Venous thromboembolism	6.1	5.9	0.012	6.0	5.7	0.014
Varicose veins	9.4	9.9	0.018	9.6	9.9	0.009
Osteoporosis	4.8	6.4	0.069	5.3	5.3	0.001
Atrial fibrillation	23.3	21.1	0.051	22.2	20.8	0.036
Fracture	1.0	1.2	0.017	1.1	1.1	0.001
**Medication (%)**†						
NSAIDs	69.0	71.4	0.054	70.0	70.9	0.021
Opioids	17.8	19.3	0.038	18.2	17.9	0.006
Antihypertensive medicine	92.9	92.6	0.010	92.6	92.1	0.021
Antidiabetic medicine	17.8	17.3	0.012	17.1	15.9	0.031
Thiazide diuretics	31.0	32.5	0.031	31.8	33.1	0.027
Nitrates	13.9	12.5	0.043	13.3	12.3	0.029
Aspirin	39.3	37.8	0.030	38.7	38.4	0.006
Loop diuretics	42.8	38.8	0.080	40.4	37.1	0.067
Potassium-sparing diuretics	15.3	12.7	0.076	13.9	12.5	0.041
Anticoagulants	19.0	17.0	0.052	18.1	17.1	0.026
Colchicine	47.8	53.0	0.103	49.9	50.5	0.013
**Healthcare utilization, mean (SD)**†						
Hospitalizations	0.6 (1.4)	0.6 (1.4)	0.018	0.6 (1.3)	0.6 (1.2)	0.029
General practice visits	9.0 (7.9)	9.3 (7.3)	0.047	9.1 (9.0)	9.0 (7.0)	0.006
Specialist referrals	0.7 (1.2)	0.7 (1.2)	0.053	0.7 (1.2)	0.7 (1.1)	0.011

* The Socio-Economic Deprivation Index was measured by the Townsend Deprivation Index, which was grouped into quintiles from 1 (least deprived) to 5 (most deprived).
† Frequency during the past year.
SMD, standardized mean difference; BMI, body mass index; n, number; y, years; SD, standard deviation; CKD, chronic kidney disease; SU, serum urate; ULT, urate-lowering therapy; eGFR, estimated glomerular filtration rate; NSAID, non-steroidal anti-inflammatory drug; TSUL, the target serum urate level.

© 2024 Wang Y et al. *JAMA Intern Med*.

eTable 3. Time-varying covariates between those who achieved TSUL through ULT in individuals with gout and CKD stage 3 and those who did not

Characteristics	Not achieving TSUL	Achieving TSUL	SMD before weighting	Not achieving TSUL	Achieving TSUL	SMD after weighting
**BMI category (%)**			0.080			0.039
Normal	14.4	16.2		14.7	14.8	
Obese	36.9	38.5		37.4	38.6	
Overweight	44.9	41.3		44.3	42.7	
Underweight	0.4	0.5		0.4	0.3	
Missing	3.4	3.5		3.2	3.6	
**Lifestyle factors**						
Drinking (%)			0.030			0.041
None	18.8	19.1		19.0	18.4	
Past	4.0	3.6		3.9	3.4	
Current	72.5	73.1		72.4	73.9	
Missing	4.7	4.2		4.7	4.3	
Smoking (%)			0.041			0.045
None	48.1	49.0		48.5	48.9	
Past	44.7	43.8		44.1	44.1	
Current	7.0	6.6		7.1	6.5	
Missing	0.3	0.5		0.3	0.5	
**Comorbidity (%)**						
Hypertension	80.0	80.5	0.012	80.3	80.5	0.005
Diabetes	28.4	27.7	0.017	27.6	26.3	0.030
Myocardial infarction	15.9	14.1	0.051	14.9	13.7	0.033
Pneumonia or infection	10.2	8.9	0.043	9.7	9.1	0.020
Dementia	1.7	1.8	0.006	1.6	1.4	0.018
Depression	10.6	11.2	0.020	10.7	10.8	0.003
Chronic obstructive pulmonary disease	10.9	10.0	0.028	10.4	9.7	0.026
Fall	15.6	16.0	0.012	15.4	14.7	0.018
Stroke	7.8	8.0	0.004	7.7	7.3	0.013
Venous thromboembolism	6.5	6.1	0.019	6.3	5.9	0.018
Varicose veins	9.5	9.9	0.013	9.7	9.9	0.004
Osteoporosis	5.3	6.7	0.061	5.6	5.6	<0.001
Atrial fibrillation	24.5	21.6	0.068	23.1	21.4	0.042
Fracture	1.8	1.1	0.006	1.8	1.7	0.003

© 2024 Wang Y et al. *JAMA Intern Med.*

**Medication (%)**						
NSAIDs	44.1	37.6	0.133	41.9	40.3	0.031
Opioids	13.4	11.7	0.052	12.5	11.4	0.033
Antihypertensive medicine	86.0	80.5	0.147	83.8	82.3	0.040
Antidiabetic medicine	17.1	15.3	0.048	15.9	14.4	0.041
Thiazide diuretics	17.8	13.9	0.106	16.6	16.3	0.008
Nitrates	11.5	8.2	0.111	10.1	8.5	0.053
Aspirin	33.9	28.7	0.113	32.0	30.7	0.029
Loop diuretics	38.9	30.8	0.169	34.9	30.7	0.091
Potassium-sparing diuretics	13.2	9.1	0.130	11.3	9.5	0.061
Anticoagulants	17.0	13.9	0.086	15.6	14.4	0.034
Colchicine	21.0	24.0	0.072	20.5	20.8	0.026

SMD, standardized mean difference; BMI, body mass index; n, number; y, years; SD, standard deviation; CKD, chronic kidney disease; SU, serum urate; ULT, urate-lowering therapy; NSAID, non-steroidal anti-inflammatory drug; TSUL, the target serum urate level.

© 2024 Wang Y et al. *JAMA Intern Med*.

eTable 4. Relationship of achieving TSUL induced by ULT to CKD progression in patients with gout and CKD stages 2-3

	Not achieving TSUL	Achieving the target serum urate level
**Severe to end-stage kidney disease**		
Number	31,066	31,066
Weighted cases (n)	1,636	1,079
Weighted mean follow-up, years	3.4	3.5
Weighted risk over five years (%)	6.14	5.02
Weighted risk difference (%, 95% CI)	0.0 (reference)	-1.12 (-2.24 to 0.00)
Weighted HR (95% CI)	1.00 (reference)	0.91 (0.82 to 1.00)*
Weighted HR from imputation (95% CI)	1.00 (reference)	0.93 (0.81 to 1.03)*
**End-stage kidney disease alone**		
Number	31,066	31,066
Weighted cases (n)	199	99
Weighted mean follow-up, years	3.5	3.6
Weighted risk over five years (%)	0.83	0.51
Weighted risk difference (%, 95% CI)	0.0 (reference)	-0.32 (-0.84 to 0.20)
Weighted HR (95% CI)	1.00 (reference)	0.73 (0.52 to 1.04)#
Weighted HR from imputation (95% CI)	1.00 (reference)	0.81 (0.56 to 1.08)$

HR, hazard ratio; n, number; 95% CI, 95% confidence interval; CKD, chronic kidney disease; TSUL, the target serum urate level; ULT, urate-lowering therapy.

* P for non-inferiority<.001

P for non-inferiority=.003

$ P for non-inferiority=.004

© 2024 Wang Y et al. *JAMA Intern Med.*

eFigure. Association of achieving TSUL achievement and CKD progression in gout patients with CKD stage 2-3

(A)

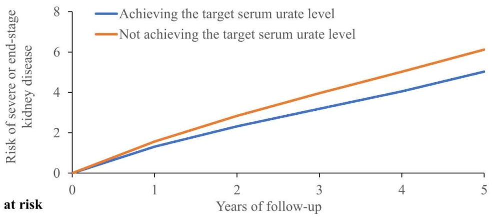

Number of replicates at risk						
**Achieving the target serum urate level**	31066	9474	8083	6805	5675	4605
**Not achieving the target serum urate level**	31066	17695	15062	12666	10530	8619

(B)

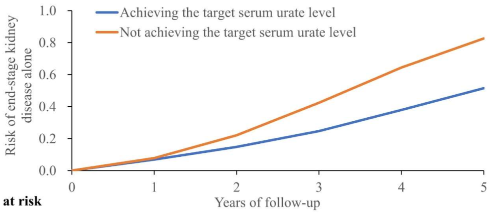

Number of replicates at risk						
**Achieving the target serum urate level**	31066	9578	8215	6979	5858	4786
**Not achieving the target serum urate level**	31066	17971	15481	13149	11036	9088

Five-year risks of severe or end-stage kidney disease (A) and end-stage kidney disease alone (B) between achieving TSULs and not achieving TSULs with urate-lowering therapy in people with gout and CKD stages 2-3. SU, serum urate; CKD, chronic kidney disease.

Abbreviations: TSUL, target serum urate level; ULT, urate-lowering therapy.

© 2024 Wang Y et al. *JAMA Intern Med*.
