# Supplementary material for: Target Serum Urate Achievement and Chronic Kidney Disease Progression in Patients With Gout and Kidney Disease
Source: JAMA Intern Med. 2024 Nov 25;185(1):74–82. doi: 10.1001/jamainternmed.2024.6212 (PMC11589860; doi:10.1001/jamainternmed.2024.6212)
Supplement: Supplement 2. — Data Sharing Statement [file jamainternmed-e246212-s002.pdf]

Data Sharing Statement

Wang. Target Serum Urate Achievement and Chronic Kidney Disease Progression in Patients With Gout and Kidney Disease. *JAMA Intern Med*. Published November 25, 2024. doi:10.1001/jamainternmed.2024.6212

Data
Data available: No
